# Building a biomedical ontology recommender web service

**DOI:** 10.1186/2041-1480-1-S1-S1

**Published:** 2010-06-22

**Authors:** Clement Jonquet, Mark A Musen, Nigam H Shah

**Affiliations:** 1 Center for Biomedical Informatics Research, Stanford University, CA 94305, USA

## Abstract

**Background:**

Researchers in biomedical informatics use ontologies and terminologies to annotate their data in order to facilitate data integration and translational discoveries. As the use of ontologies for annotation of biomedical datasets has risen, a common challenge is to identify ontologies that are best suited to annotating specific datasets. The number and variety of biomedical ontologies is large, and it is cumbersome for a researcher to figure out which ontology to use.

**Methods:**

We present the *Biomedical Ontology Recommender web service*. The system uses textual metadata or a set of keywords describing a domain of interest and suggests appropriate ontologies for annotating or representing the data. The service makes a decision based on three criteria. The first one is *coverage*, or the ontologies that provide most terms covering the input text. The second is *connectivity*, or the ontologies that are most often mapped to by other ontologies. The final criterion is *size*, or the number of concepts in the ontologies. The service scores the ontologies as a function of scores of the annotations created using the National Center for Biomedical Ontology (NCBO) *Annotator web service*. We used all the ontologies from the UMLS Metathesaurus and the NCBO BioPortal.

**Results:**

We compare and contrast our Recommender by an exhaustive functional comparison to previously published efforts. We evaluate and discuss the results of several recommendation heuristics in the context of three real world use cases. The best recommendations heuristics, rated ‘very relevant’ by expert evaluators, are the ones based on coverage and connectivity criteria. The Recommender service (alpha version) is available to the community and is embedded into BioPortal.

## Introduction

### Background

Biomedical ontologies are widely used to design information retrieval systems, to facilitate interoperability between data repositories, and to develop systems that parse, annotate or index biomedical data resources. Biomedical researchers use ontologies and terminologies to annotate (or tag) their data with ontology terms for better data integration and translational discoveries [1,2]. The number and variety (formats, locations) of biomedical ontologies is now so large that choosing one for an annotation task or for designing a specific application is a difficult challenge. Besides, re-usability is a desired practice in ontology development both because the process of building an ontology from scratch is long and hard and because the community needs to avoid the multiplication of several competing ontologies to represent similar knowledge.

However, the process to choose a set of ontologies to use is oftentimes a hard, manual and time consuming task for researchers. Members of the National Center for Biomedical Ontology (NCBO) often get requests for suggesting an appropriate ontology for a certain domain or application. There are several uses cases for ontology recommendation:

• Re-use existing ontologies when constructing new ones;

• Identify the most appropriate ontology for a given domain;

• Support an annotation workflow.

Researchers lacking an appropriate ontology may need to reprocess or re-annotate their data or redesign their application later. They may also start to develop a new ontology instead of re-using a standard shared one. They may also miss insights they might have seen had they used the right ontology when integrating their datasets with other datasets [6].

Therefore, ontology recommendation has emerged has a key issue in biomedicine. The manner in which recommendation occur depends on user settings. In some cases, the recommendation process can be long and non-automatic; the user can participate in the process (e.g., answer questions to refine the query) to enhance the accuracy of results. In other cases, a quick and fully automated approach is required, such as when ontology selection occurs at runtime in an application. For example, Sabou et al. [3] presented the requirements of a semantic browsing application called *Magpie*. Magpie needs to identify the ontologies that offer maximum coverage of a web page topic in order to identify the concepts in the web page and provide users with related information. As another example in the biomedical domain, *Reflect *[4] recognizes and highlights gene, protein and small molecule names while browsing a web page. For each recognized entity, Reflect provides a description, related information and links such as to PubMed abstracts. Both Magpie and Reflect need an ontology selection approach; however, Magpie requires a fully dynamic and automatic method that must be called at runtime, whereas Reflect requires preselection of the ontologies or vocabularies to use during application design.

This paper focuses on providing a quick automated recommendation with minimal user burden. We considered two main recommendation scenarios differentiated by the type of input provided by the user:

• *Corpus-based recommendation*: Given a corpus of textual metadata describing some elements of a biomedical dataset, our system recommends appropriate ontologies to annotate the dataset with ontology concepts.

• *Keyword-based recommendation*: Given a set of keywords/terms representative of a domain of interest, our system recommends appropriate ontologies to consider for re-use or extension for researchers building new ontologies or semantic applications.

The related works show that these two scenarios are the most frequent in the ontology recommendation literature. They have important differences. For example, the keyword-based recommendation is potentially cleaner because it avoids the introduction of spurious terms. However, it cannot account for term frequency. If a term appears several times in a corpus, one may want to give to the ontology that contains the corresponding concept a better score as this ontology is more relevant to the domain of the corpus.

### Contribution

This paper describes the *Biomedical Ontology Recommender web service*, or *Recommender*. Given textual metadata or a set of keywords describing a domain, the Recommender suggests ontologies appropriate for annotating or representing the data. Sabou et al. [3] demonstrate that a single ontology rarely provides the complete coverage or application need. Therefore, ontology selection systems need to be able to return combination of ontologies as result.Appropriateness is evaluated according to three main criteria:

• Coverage: the ontology that best covers the given data;

• Connectivity: the ontology containing the terms that are most often mapped (or referred) to by other ontologies;

• Size: the number of concepts in the ontology.

To facilitate and encourage the annotation of biomedical datasets, we created the *NCBO Annotator Web Service *[5], which annotates textual data with ontology concepts. We call annotation a mapping between a textual data and an ontology concept that declares: particular data “is associated with” a particular concept. The *Annotator* scores each annotation based on whether the term found is a preferred name, synonym, ancestor term or mapped term of a concept mentioned in the text. Biomedical researchers can use the Annotator to automatically tag datasets with ontology concepts. For example, the Gminer project (http://gminer.mcw.edu) used it to annotate rat microarray experiments. We used it to index public biomedical data resources with ontology concepts [6].

The Annotator uses one of the largest available sets of biomedical ontologies including the NCBO BioPortal ontologies and the Unified Medical Language System (UMLS) Metathesaurus ontologies. The NCBO BioPortal [7] is a web repository of biomedical ontologies. Users can browse, search, and comment ontologies both online and via a web services application programming interface. The UMLS Metathesaurus [8] is a collection of concepts, terms and their relationships from various biomedical controlled vocabularies, terminologies and ontologies.

This study describes a use of the Annotator service to implement the Recommender service. We present recommendation heuristics for suggesting ontologies in corpus & keyword-based recommendation scenarios. They aim to address the following questions:

1. Which ontologies offer maximum coverage for a set of data?

2. Which ontologies are reference ontologies for a set of data?

3. Which small ontologies are specialized for a set of data?

We evaluate and discuss recommendation results generated by each heuristics in the context of three real world use cases. The best recommendation heuristics, which address both questions 1 and 2, were rated ‘very relevant’ and ‘relevant’ by expert evaluators.

An alpha version of the Recommender is publicly available and described at http://www.bioontology.org/wiki/index.php/Ontology_Recommender_Web_service. It is deployed as a REpresentational State Transfer (REST) web service for programmatic access. It can also be accessed through a user interface. The Recommender service is currently being moved into a production environment and embedded in the BioPortal web application. Additional file 1 provides documentation describing how to use the service.

## Methods

**Figure 1:**
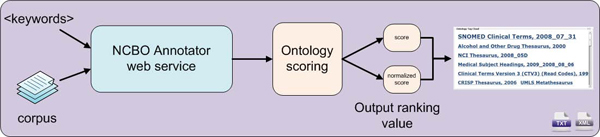
Recommender service workflow.

Figure 1 describes the overall workflow of the Recommender. The service accepts biomedical text data as input and suggests the most appropriate ontologies. The annotations used to generate the recommendation are produced by the Annotator summarized in the next section. Next, according to the annotations, ontologies are scored and ranked with different output values. Results can be returned as text or XML.

### NCBO Annotator web service

**Figure 2:**
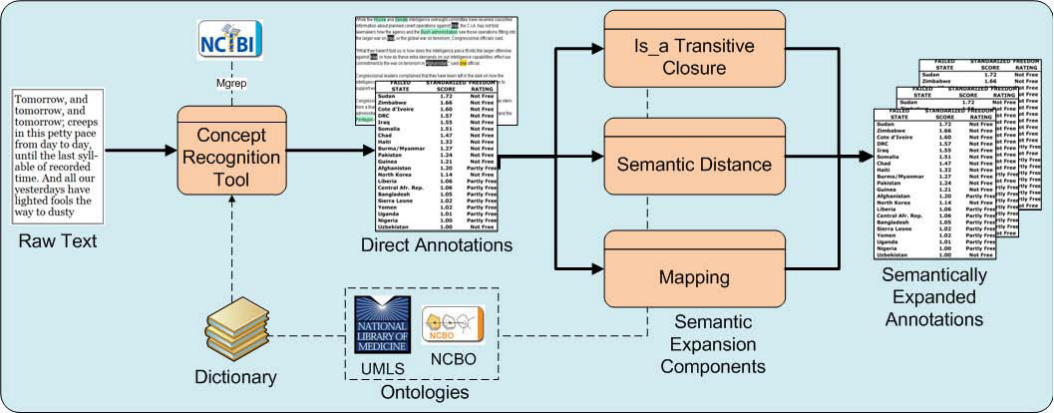
**NCBO Annotator web service workflow.***Direct annotations are created from raw text based on syntactic concept recognition (concepts names & synonyms). Next, different components expand the first set of annotations using the knowledge represented in one or more ontologies.*

The Annotator web service workflow is composed of two main steps (Figure 2) [5]. First, direct annotations are created from raw text. Annotations are based on syntactic concept recognition using a dictionary compiled from terms (concept names and synonyms) pulled from the ontologies. The Annotator enables the selection of ontologies from one of the largest sets of available biomedical ontologies. We implemented the service using the 98 English ontologies in UMLS 2008AA and a subset of the BioPortal ontologies (122 as of this writing). These ontologies provide a dictionary of 4,222,921 concepts and 7,943,757 terms. In the second step, semantic expansion components leverage the semantics in ontologies (e.g., *is_a* relations and mappings) to create additional annotations. For example, the *is_a transitive closure* component traverses an ontology parent-child hierarchy to create new annotations with parent concepts of concepts in direct annotations. The *ontology-mapping* component creates new annotations based on existing mappings between different ontologies. Point-to-point mappings that link concepts one another are defined manually or by automatic algorithms in the UMLS Metathesaurus and in BioPortal.

We used the results of the Annotator to score ontologies. When using the Recommender, users can use the UMLS ontologies, the BioPortal ontologies, or both. By annotating user data with all available ontologies, we computed statistics and compared the ontologies to one another to recommend the most appropriate ones. We used the Annotator with two possible settings (see the appendix for exact parameters):

• Concept recognition only (CR);

• Concept recognition + mapping expansion (CR+M).

The concept recognition step (CR) allows the Recommender to evaluate an ontology following the *coverage* criterion. Ontologies containing more concepts mentioned by name or with synonyms in text data will create more annotations. Activating the mapping expansion (CR+M) has two interesting effects:

• *It extends coverage to terms defined in other ontologies*. For example, if the word ‘treatment’ is passed to the Annotator without mapping expansion, a direct annotation with the concept MSH/C0087111 (*treatment* in MeSH) is identified, but no annotations are identified in SNOMED-CT. This is because the term ‘treatment’ does not exist in that ontology. However, with mapping expansion, a UMLS point-to-point mapping (based on CUI) MSH/C0087111->SNOMEDCT/C0087111 can be used to generate an expanded annotation with concept SNOMEDCT/C0087111 (*therapeutic procedure* in SNOMED-CT). Therefore, SNOMED-CT could also be considered as a potentially good ontology for text data containing the word ‘treatment.’

• *It gives importance to reference ontologies i.e., ontologies that are good destinations for mappings (connectivity criterion).* The mapping to an ontology by many other ontologies shows its popularity and importance in the domain. For example, if the word ‘melanoma’ is passed to the Annotator without mapping expansion, two direct annotations are identified with 40644/Melanoma (*melanoma* in NCI Thesaurus) and 40465/DOID:1909 (*melanoma* in Hunan Disease). However, with mapping expansion, an expanded annotation is also generated with 40644/Melanoma using a mapping defined by a user in BioPortal 40465/DOID:1909->40644/Melanoma. In this way, the Recommender gives the NCI Thesaurus more importance. In a previous study [9], we demonstrated the existence of hub ontologies in the network of biomedical ontologies. Hubs are connected one another by point-to-point mappings. For example: SNOMED-CT, the National Drug File Reference Terminology, MeSH, and the NCI Thesaurus are hubs. Our study demonstrated that 33% of ontologies have at least half of their concepts mapped to concepts in other ontologies.

Annotations are scored according to the context from which they were generated (direct concept recognition or semantic expansion) and returned to the user. The annotation scoring method is detailed in next section.

We did not consider *is_a* transitive closure expansion for recommendation because it gives more importance to ontologies with multiple inheritances. Examining ontology structure in order to discriminate ontologies has been suggested [10] and is discussed later in this paper.

The Annotator service provides a 'longest only' parameter to refine the matches to ontology concepts. If longestOnly=true, the Annotator selects only the longest term matching phrase. For example, if longestOnly=true, the phrase 'breast cancer' generates only ‘breast cancer.” If longestOnly=false, it generates three annotations: 'breast', 'cancer' and 'breast cancer.' The way the Annotator behaves with this parameter is even more useful for discriminating ontologies from one another. In fact, if it finds an annotation with the complete phrase in an ontology composing the dictionary, partial annotations with other ontologies will not be generated. For example, because 'breast cancer' exists in Human Disease and the NCI Thesaurus, if longestOnly=true, annotations generated with those terms will block annotations with the terms 'breast' in the Vaccine Ontology or 'cancer' in BIRNLex. This feature is interesting for the Recommender, as it allows enhancing the appropriateness of Human Disease and the NCI Thesaurus for the given phrase.

### Scoring method and output values

The score is a number assigned to an annotation to indicate its importance. Higher scores reflect more important annotations. The scoring algorithm gives a specific weight to an annotation according to its context, as well as matching terms. For instance, an annotation done by matching a concept’s preferred name gets a higher weight than one done by matching a concept’s synonym or one done with a parent-level-3 (ancestor) concept in the *is_a* hierarchy. In the previous example that considered the word 'treatment,' the Annotator would give more importance to ontologies containing the term 'treatment' than to ones containing the term 'therapeutic procedure.' Table 1 describes the weights used by the scoring algorithm.

**Table 1:** Annotation weights per context

Annotation context	Weights
Direct annotation done with a concept preferred name	10
Direct annotation done with a concept synonym	8
Expanded annotation done with a mapping	7
Expanded annotation done with a parent level n(e.g., 9 for n=1; 7 for n=2; 4 for n=5; 3 for n=8; 1 for n>12)	1+10.e^-0.2*n^

The Recommender service uses the outputs of the Annotator to rank ontologies according to two output values:

1. *Score*: the sum of the scores of all the annotations generated with concepts from a particular ontology;

2. *Normalized score*: the score divided by the ontology size.

Each output ranking value is expected to provide different results in the scenarios considered. The score value is appropriate for the corpus-based recommendation, as it reflects the importance of terms appearing several times in the corpus. The normalized score is expected to help users distinguish between large ontologies that offer very good coverage of input data and small ontologies with correct coverage, yet more specialized to the input data’s domain. Without assuming that small ontologies are better defined/formalized than larger ones, we assumed that this information could be of value to users.

Table 2 summarizes the questions we defined in the Introduction and the heuristics to address them.

**Table 2:** Recommender’s heuristics and corresponding research questions.

Annotator’s method	Output value	Question
CR	score	Which ontologies offer maximum coverage for a set of data?
CR+M	score	Which ontologies are reference ontologies for a set of data?
CR	normalized-score	Which small ontologies are specialized for a set of data?

### Example

Consider the text: *“Melanoma is a malignant tumor of melanocytes which are found predominantly in skin but also in the bowel and the eye.”* Sent to the Annotator, this sentence generates the following direct annotations—i.e. string matching with dictionary. (The numbers in the curly braces give the annotation weights):

• NCI/C0025201, *Melanocyte* in NCI Thesaurus {10}

• NCI/C0025202, *Melanoma* in NCI Thesaurus {10};

• NCI/C0027651, *Neoplasm* (synonym of *tumor*) in NCI Thesaurus {8};

• FMA/C0015392, *Eye* in FMA {10}

• FMA/C0021853, *Intestine* (synonym of *bowel*) in FMA {8}

• 40465/DOID:1909, *Melanoma* in Human Disease {10};

The mapping expansion generates the annotation (thanks to UMLS mappings):

• FMA/C0025201, *Melanocyte* in FMA, concept mapped to NCI/C0025201 {7}.

• NCI/C0015392, *Eye* in NCI Thesaurus mapped to FMA/C0015392 {7}.

The final score, using CR+M, is the sum of the annotations score per ontology:

• NCI Thesaurus (NCI): 35

• Foundational Model of Anatomy (FMA): 25

• Human Disease (40465): 10

**Figure 3:**
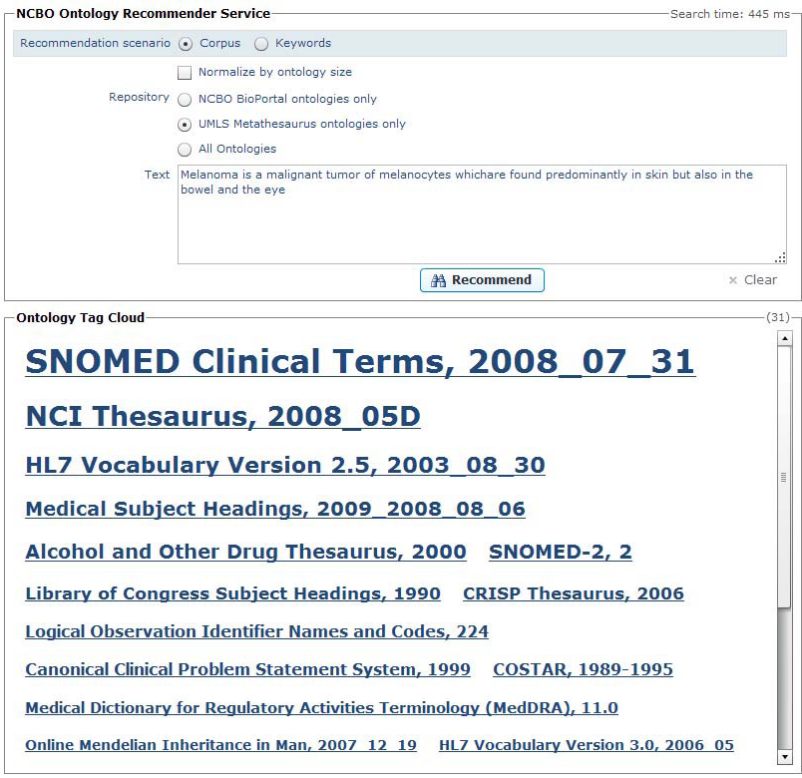
**Recommender web service user interface.***A user can select the recommendation scenario, as well as the repository of ontologies to use, and enter the text data to recommend. A tag cloud is generated in which the score of an ontology is represented by the size of its name in the cloud.*

Figure 3 shows results for this text in the Recommender service user interface (UMLS ontologies only). SNOMED-CT is the highest scored ontology.

## Results and evaluation

In this section we present the results of a use case-based evaluation of the Recommender service. We asked three different groups to provide us with a corpus and a set of keywords representing the data they would like to use ontologies for. These evaluators were knowledgeable about biomedical ontologies and have already experienced the process of selecting the ontologies appropriate for their task. Thus, they were well-suited to evaluating the utility of the Recommender service for their datasets. Table 3 shows the source & size (number of words) of the datasets provided by each group. For each dataset, we ran the Recommender with the two methods (CR & CR+M) and generated rankings with two values (*score* and *normalized score*).

**Table 3:** Source and size of the six datasets.

Dataset	Source	Size
**UC1-keyword**	Provided by evaluator	420
**UC1-corpus**	Methods section of 3 papers about ECG-related paper	2750
**UC2-keyword**	Provided by evaluator	9615
**UC2-corpus**	Concatenated ‘name’, description’ and ‘species’ sections of 30 randomly selected ArrayExpress entries	6520
**UC3-keyword**	Provided by evaluator	72
**UC3-corpus**	National Comprehensive Cancer Network (NCCN) Breast Cancer Guideline	12540

### Use cases

UC1: Researchers at the Center for Cardiovascular Bioinformatics and Modeling Johns Hopkins University developed an electrocardiogram (ECG) ontology. This ontology describes ECG data collection protocols, features of time-evolving ECG waveforms, ECG analysis algorithms, and data derived from ECG analyses. Its main role is to enable cardiovascular researchers to share and analyze primary and derived ECG annotated data. These researchers were interested in maximizing re-use of existing ontologies when building the ECG ontology.

UC2: Researchers at the European Bioinformatics Institute (EBI) developed an ontology focused on modeling experimental factors in the ArrayExpress database (http://www.ebi.ac.uk/microarray-as/ae/). The ontology was developed to “increase the richness of the annotations that are currently made in the ArrayExpress repository, to promote consistent annotation, to facilitate automatic annotation and to integrate external data.” These researchers wished to map their new ontology to existing domain-specific ontologies.

UC3: Researchers at Stanford University are building a system that abstracts clinical information from two electronic medical record databases related to the care and management of breast cancer. Their goal is to assess quality of care and adherence to clinical guidelines as described in the National Comprehensive Cancer Network (http://www.nccn.org). These researchers wished to reuse ontologies that have been developed by other organizations to build their application.

### Results

To evaluate the recommendation produced for each dataset, we asked the evaluators for a reference ranking i.e., the ten ontologies that they would have liked to obtain. We also asked the evaluators to comment on our Recommender’s ranking and to give it a mark between 1 and 5:

• *Very relevant* (5) – The recommendation exactly matched the results of the researcher’s investigation, and the top-ranked ontologies were the ones appropriate for their data. The ranking is almost the one suggested by the researchers.

• *Relevant* (4) – The recommendation provided useful information. Most of the top ontologies were relevant. The ranking was fair.

• *Correct but not really relevant* (3) – The recommendation was technically correct but did not really help the researcher select the most appropriate ontologies. Some top ontologies were relevant others were not.

• *Few relevant* (2) – The recommendation was useless, or few relevant ontologies were identified.

• *Not relevant* (1) – The recommendation was wrong. The top-recommended ontologies were obviously not relevant.

Considering the large number of ontologies (220) that the Recommender uses and considering the presence of 5 duplicates (e.g., NCI Thesaurus being present both in UMLS (RRF format) and in BioPortal (OWL format)) we asked the evaluators to examine our top 15 results to evaluate a recommendation. Table 4 presents their evaluations.

**Table 4:** Evaluation of Recommender results.

Method	Output	UC1-key-word	UC1-corpus	UC2-key-word	UC2-corpus	UC3-key-word	UC3-corpus
**Concept recognition only (CR)**	Score	5	4.5	4	3	5	5
	Normalized score	4.5	4.5	4	2	2	1

**Concept recognition + mapping (CR+M)**	Score	4	4	3	4	4.5	4.5
	Normalized score	3.5	4	2.5	2	1	1

### Results analysis

Overall, the evaluators were positive about the utility of the Recommender service. They all agreed that it would have helped them select ontologies for their task. On examining the results and their comments and marks, the following observations stand out:

• With the score output, large ontologies (17% of ontologies are above 20K concepts) were easily correctly identified. The high number of concepts in those ontologies makes them more appropriate for fully marking up or tagging textual descriptions. The large ontologies were usually among the top ten ranked ontologies. For example, SNOMED-CT (313K concepts & 972K terms), MeSH (291K concepts & 682K terms), Clinical Terms - Read Codes (186K concepts & 347K terms), and the NCI Thesaurus (74K concepts & 183K terms) were often in the results. We note that the NCI Thesaurus, which is not the largest ontology, was in the top 3 results for all the datasets.

• With the score output, moderately sized ontologies (36% of ontologies are between 1K & 20K concepts) and small ontologies (47% of ontologies are <1K concepts) were less often correctly identified. For example, the Recommender found Experimental Factor Ontology (2406 concepts & terms) in UC2-keyword or Human Developmental Anatomy (8340 concepts & terms) in UC1-keyword. But the Recommender missed some ontologies, regardless of the method used. For example, it never identified the Ontology for Biomedical Investigations in UC1 or RxNorm in UC3 whereas those ontologies were expected by the evaluators.

• Overall, the score was more informative than the normalized score. Normalization with ontology size placed importance on small ontologies containing a few terms in the dataset. Sometime, as in UC3, this feature was not considered relevant due to the introduction of considerable noise (e.g., Amino Acid (46 concepts & terms)). Sometime this feature is relevant, as in UC1, where it allows identifying 7 or 8 new ontologies missed by the score ranking such as Spatial Ontology (109 concepts & 168 terms), Ontology of Homology and Related Concepts in Biology (65 concepts & 132 terms). The Electrocardiography Ontology (497 concepts & terms) was also one of them. This result was particularly relevant as this is the ontology developed at Johns Hopkins University. In UC2, the Recommender correctly indentified Common Anatomy Reference Ontology (46 concepts & 50 terms) and Experimental Factor Ontology (2406 concepts & terms). This result was also particularly relevant as this ontology was specifically developed at EBI to annotate ArrayExpress experiments.

• The intersection of the top ten ontologies obtained with the score output and the top ten ontologies obtained with normalized-score output was small. We note that, the ontologies in this intersection are generally very relevant to the dataset; for example, Experimental Factor Ontology or Uber Anatomy Ontology in UC2 or Mass spectrometry in UC1.

• The normalized score is more informative for keyword-based recommendations than with corpus-based recommendation. This result is not surprising, because in a keyword-based recommendation, the score is supposed to be directly proportional to the number of unique concepts identified in the keywords. Therefore, ranking after normalizing the score is equivalent to ranking based on percentage of overlap between the keywords and the ontology.

• The influence of the mappings was noted by the evaluators as resulting in giving disproportionate importance to reference ontologies. Indeed, the use of the mappings will slightly change the ranking of the top ontologies that can help distinguishing between large ontologies (which are often actually the ones with many mappings). However, this was not the preferred ranking for most uses cases (except UC2-corpus). Furthermore, the use of mappings does not help in identification of average size and small ontologies.

• The influence of mappings was in fact detrimental in the keyword scenario. In this scenario, where a user provided the exact terms to map to ontology concepts, the activation of mappings introduced noise.

• The influence of mappings was not significant after results were normalized by ontology size.

• The size of the dataset influenced recommendation quality. Indeed, the smallest datasets received the lowest marks (e.g., UC1-keyword and UC3-keyword). This finding is expected because the more data the Recommender has, the better the recommendation will be.

• We note that when a significant and a large enough set of keywords is provided e.g., UC2, the recommendations generated based on keywords are preferred over the ones generated from corpus data. This is also an expected behavior because corpus data introduces spurious text phrases that bias the results. Especially, we note that the rankings based on the normalized score are significantly better with a large set of keywords.

• When used without the mappings expansion (CR), we obtained an average response time of 15 seconds for 1000 words with the service’s current implementation. However, activating the mapping expansion (CR+M) slows its performance to ~1 hour for 1000 words.

The original datasets used as well as the recommendations generated are available at: http://www.bioontology.org/wiki/index.php/Ontology_Recommender_Web_service

## Related work

**Figure 4:**
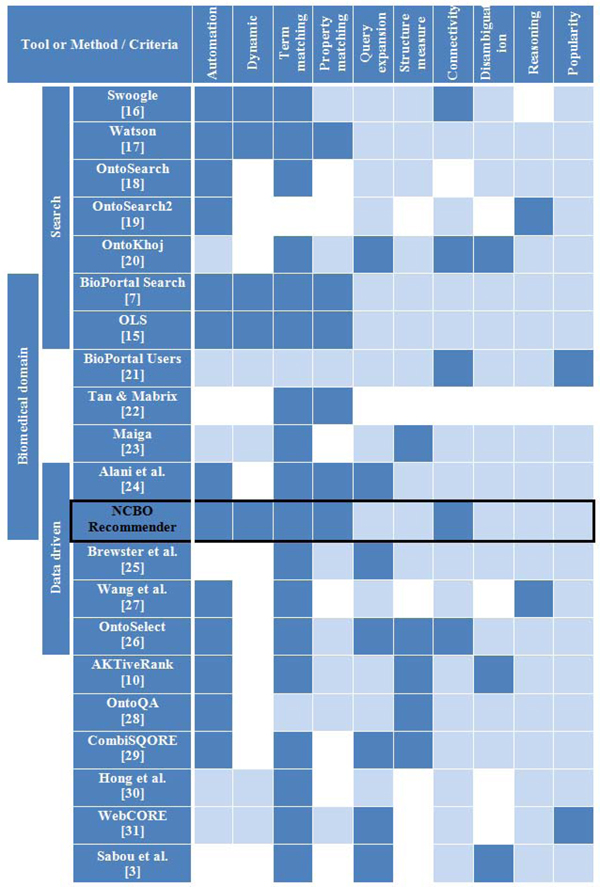
**Comparison of ontology selection approaches.** ***Bold blue filled cells mean a positive value for the given criterion whereas light blue cells mean a negative value. White cells are undetermined or not applicable.***

Much work has been done in the semantic web community on evaluating the quality of an ontology for a particular criteria or for a specific application. In this section we present a summary of the recent literature about ontology recommendation. We identified 20 tools or methods and compared them from high level functional perspective described below and summarized in Figure 4 (see additional file 2 for a detailed description of each tool and method). Only a few approaches (6) are applied to the biomedical domain.

### Recommendation criteria

We performed a functional comparison for the different tools or methods identified in the literature based on the following criteria:

• **Automation**: *Is the tool or the method fully automatic or does it requires an interaction with the user for whom a recommendation is necessary?* For examples, [30,20,31] are non-automatic method in which users are involved in the recommendation process. In OntoKhoj the user is requested for the disambiguation process.

• **Dynamic**: *Is the tool quick enough to be dynamically invoked by client applications and accurate enough to avoid requiring human intervention to clean the results?* For example, AKTiveRank can be dynamically invoked only if the ontology returned by the underlying search engine has already been processed once; otherwise the response time is strongly affected. Non-automatic approaches are excluded from this criterion. Part of this criterion is called ‘run time performance’ in [11].

• **Term matching**: *Is the tool or method based on any kind of matching between the query terms (directly submitted, or expanded, or from a corpus) and the class and property names of the ontologies?* This kind of matching could be exact match, or fuzzy match (e.g., contains, stemming) as in [3]. This criterion is called ‘class match measure’ in AKTiveRank or ‘coverage’ in OntoSelect or ‘topic coverage’ in [11].

• **Property matching**: *Does the tool or method exploits any kind of matching between the query terms and the property values of the classes e.g., definition, synonyms?* For instances, [24], BioPortal Search and OLS do matching using the concept names and synonyms as the Recommender does. Being restricted to a specific domain does facilitate such feature implementation as it is easier to specify which property values to look up into.

• **Query expansion**: *Does the tool performs any form of query expansion to retrieve a more representative set of terms to match with the ontologies?* For examples [20,25,3,31] use WordNet to expand the query terms with hypernyms, hyponyms or synonyms. As another example, [24] expands the user query by automatically obtaining a corpus for the given the keywords via Google or Wikipedia.

• **Structure measure**: *Is the tool or method based on some formal measures of the ontology structure?* This criterion is similar to ‘richness of knowledge’ in [11] or ‘structure’ in OntoSelect. AKTiveRank proposes two of such measures: the ‘centrality measure’ based on the position of the matching concepts in the hierarchy (i.e., middle level concepts are given more importance; the ‘density measure’ based on the number of relation for a concept (i.e., concepts with high numbers of subclasses, superclasses and instances are given more importance). [23] and [28] also propose a granularity measure based on relation richness.

• **Connectivity**: *Does the tool or the method exploits the possible references (e.g., import, instantiation) or link (e.g., mappings) between ontologies in order to give more importance to reference ontologies?* This criterion is called ‘popularity’ in [11] or ‘connectedness measure’ in OntoSelect. For example the PageRank approach of Swoogle and OntoKhoj.

• **Disambiguation**: *Does the tool or the method performs any kind of disambiguation or semantic matching using the semantics of the ontology when doing the matching with terms?* For example, AKTiveRank measures the ‘semantic similarity’ between matching concepts and ontologies in which matching concepts are semantically close to one another are better ranked. OntoKhoj includes a disambiguation process involving the user.

• **Reasoning**. *Does the tool or method uses any kind of reasoning?*

• **Popularity**: *Does the tool or method uses any kind of users direct (e.g., reviews, notes) or indirect (e.g., usage logs) assessments to rank ontologies?* For examples, reviews & notes entered in BioPortal by users or assements used in WebCORE.

The notion of *coverage*, previously mentioned in the paper can now be described as the conjunction of the term matching and property matching.

### Summary of the limiations of current tools for application to biomedical ontologies

In their study Sabou et al. [11] identified two major shortcomings of ontology selection approaches:

• Relations between concepts are ignored most of the time;

• The meaning of concepts is ignored most of the time.

In light of the comparison done in Table 5, the former shortcoming is not valid anymore as eight methods address the ‘structure measure’ or ‘connectivity’ criteria. However, the latter shortcoming still stands because ‘property matching’ or ‘disambiguation’ criteria are not well addressed; probably because correctly identifying ontology concepts in keywords or unstructured text is still a hard task. For example, if a user submits only the term ‘cold’ to an ontology selection system, it is impossible to figure out if the intent of the user is to get results for the disease or for the sensation. This is an issue that some methods propose to address either by interaction with the users or by having a corpus-based approach. The analysis of the literature explains the need of a new tool for biomedical ontology recommendation; particularly because:

• Many methods are not implemented into a concrete application or service that can actually be used by the biomedical community;

• The number of available ontologies is often limited;

• Few of the tools handle biomedical ontologies in non semantic web standard format (e.g., OBO, RRF);

• Despite the limited use of meaning of concepts, simple properties such as synonyms, which are often not available in a general context, are usually explicitly defined in biomedical ontologies;

• The used of mappings is missing. Resources like the UMLS Metathesaurus or NCBO BioPortal are now very rich in point-to-point mappings [9];

• Only one method suggests using the size of the ontology which according to the results presented before could sometime be relevant.

**Table 5:** Addressing of each questions (automation, speed and accuracy).

Question – Recommender’s method	Automation	Fast enough	Accuracy
Which ontologies offer the maximum coverage for my data? – (CR – score)	Yes	Yes	Yes
Which ontologies are reference ontologies for my data? – (CR+M – score)	Yes	No	Yes
Which small ontologies are specialized for my data? – (CR – normalized score)	Yes	Yes	Not enough

All these limitations are addressed by the Recommender in a simple web service application that can recommend from over 220 biomedical ontologies.

### Discussion and future work

The task of recommending an ontology is hard because of the variety of user requirements and expectations: “Good ontologies are the ones that serve their purpose” [25]. The perfect recommendation method, automatic, easy-to-use, and completely accurate does not probably exist. The related work illustrates the importance of being able to address the two scenarios of corpus & keyword based recommendation. The analysis of the results demonstrates that the Recommender successfully addresses both scenarios. Human intervention is needed to clean the noise introduced in the recommendation when normalizing by ontology size (i.e., third question). Moreover, the response times obtained with the CM method are good enough to envision embedding the Recommender into software application e.g., semantic browsing. Table 5 summarizes questions addressed by the Recommender.

When analyzing the results, one can say that normalizing by the size is not enough to identify specialized ontologies. However, discriminating ontologies based on the ontology structure (as suggested in related work) is hard and subjective i.e., how to decide to give more importance to formalism rather than to another one. This is the reason why we do not use the Annotator is_a transitive closure expansion.

Also, the mapping expansion appeared useful but its influence was offset by coverage. In the future, we would like the Recommender to abstract on service configurations (e.g., context weights, parameters, criteria) in order to enable further control on the scoring routine. Each user can therefore select the ‘scenario’ that matches the best his needs. For instance, a user with a small set of keywords would prefer to use the corpus-based recommendation; whereas a user with a large set of keywords would rather go for the keyword recommendation scenario.

We also note that the results of the Recommender are dependent on the accuracy of the NCBO Annotator, which uses lexical matching for concept recognition and the limitations that go with it [5]. Matching text to ontology concepts is a hard task. A major drawback of ontology selection approaches is to be based on some kind on lexical matching between concepts and keywords. The matching methods do not take into consideration the semantics of the ontologies when doing the look up. The Recommender service provides two useful features to partially address this issue:

• The services uses synonyms, because synonyms are the first step to accomplish a semantic match.

• The service uses mappings between ontologies to leverage semantics that has been implied by connecting ontologies together.

We note that the recommendation could be greatly enhanced if the annotations could be scored according to a degree of specificity of the concept forming the annotation. Indeed, concepts like ‘cancer’, ‘cell’ or ‘disease’ are less specific than ‘pheochromocytoma’ or ‘appendicectomy’. One might want to see the ontologies that contain the specific terms, ranked higher than the ones containing the less specific ones. Evaluating concept specificity is indeed a challenging task. Within the context of BioPortal, we are investigating three different approaches: (i) based on the usage (e.g., to mine the user logs); (ii) based on the usefulness (annotated data); (iii) based on the ontology structure (e.g., to use the neighborhood/relationships of a concept to determine a degree of importance).

Furthermore, the recommendations are often linked to the size of ontologies. Large ontologies are easier to identify than small ones. The ontology recommendation challenge has to reconcile two conflicting effects related to the size of ontologies: On one hand, large ontologies have the advantage of a large coverage that allows good reusability and data integration. However, these ontologies are hard to manipulate as they introduce noise (e.g., numbers or country in SNOMED-CT) and are sometime hard to process (e.g., memory loading, reasoning, search). On the other hand, small ontologies are easily usable and processable and are adequate for precise tasks. However, these ontologies are sometime too specialized in order to be re-usable and lack links to other ones that will facilitate data integration. The result is that the most often users want to deal with a small piece of a large ontology i.e., an ontology view (or module). This is, indeed, the hardest thing to do. To the best of our knowledge there is no tool or method that has addressed the question of recommending only an ontology view or a subpart of an ontology. This challenge is also identified in Sabou et al. in [11,3]. With the introduction of some type of ontology views (e.g., hierarchy branch) in BioPortal [21], it would be interesting to extend the Recommender service with the possibility to recommend views.

Finally, the evaluators mentioned the requirement to use the Recommender with a customized list of ontologies. It appears that in some cases, users already know the ontologies to use in their task. In those cases, users are not interested in the “recommendation/selection” aspect but in the “evaluation” aspect provided by the Recommender (cf additional file 2). Undeniably, the noise introduced in the recommendations by the other ontologies makes the evaluation process a bit harder. We are currently implementing a way to filter the recommendations per ontology in order to address those uses cases.

## Conclusions

Biomedical ontologies have been identified as a crucial means for representing knowledge and annotate biomedical data in order to create a biomedical semantic web. Ontologies facilitate data integration and translational discoveries[6]. In this paper, we have discussed the need of ontology recommendation in order to design new ontologies and annotate data. We have presented the *Biomedical Ontology Recommender web service*, which – given textual metadata (corpus or keyword describing the domain of interest) – suggests appropriate ontologies relevant for annotating the given data. Our approach uses both a syntactic concept recognition step (string matching with concept names & synonyms) and a mapping expansion step to enforce reference ontologies (expand annotations with point-to-point mappings). The Recommender uses over 220 ontologies from the UMLS Metathesaurus and the NCBO BioPortal, which is to the best of our knowledge, unique in biomedicine. We have demonstrated the Recommender’s performance on several recommendation heuristics via a use case-based evaluation. Overall, evaluators agreed on the utility of the recommendations provided both for their keyword and corpus datasets. We have compared and contrasted our approach with other methods and tools that have been published in the literature. We have also identified the key outstanding issues in ontology recommendation that point to fruitful research directions; such as: (i) selecting small and specialized ontologies; (ii) semantically matching free text and ontology concepts; (iii) recommending according to a specific desired criterion; (iv) recommending ontology views. In the future, we expect further work on the Recommender service to address those issues.

## Competing interests

The authors declare that they have no competing interests.

## Authors' contributions

CJ designed the Recommender workflow, implemented the web service and wrote the manuscript. NHS conceived of the project, participated in technical discussions as well as system design, and edited the manuscript. MAM supervised and mentored CJ and NHS.

## Appendix

### Parameters to give to the NCBO Annotator web service

The following NCBO Annotator parameters are used to implement the Recommender service (non specified parameters are set to default values):

**Table 6:** 

longestOnly	= true	[for keyword-based recommendation]
	| false	[for corpus-based recommendation]
withDefaultStopWords	= true	
minTermSize	= 3	
localSemanticTypeIDs	= T000	[for UMLS Metathesaurus repository]
	| T999	[for NCBO BioPortal repository]
	| nothing	[for both repositories]
activateMapping	= true	[for CR+M method]
	| false	[for CR method]

## Supplementary Material

Additional file 1 Description of data: Contains the detailed documentation on how to use the Recommender web service.
File format: Microsoft Word 97-2003 Click here for file

Additional file 2 Description of data: Contains a detailed comparison of the existing ontology recommendation tools that form the basis of the related work section.
File Format: Microsoft Word 97-2003 Click here for file
