# Supplementary material for: Building a biomedical ontology recommender web service
Source: J Biomed Semantics. 2010 Jun 22;1(Suppl 1):S1. doi: 10.1186/2041-1480-1-S1-S1 (PMC2903720; doi:10.1186/2041-1480-1-S1-S1)
Supplement: Additional file 1 — Description of data: Contains the detailed documentation on how to use the Recommender web service. File format: Microsoft Word 97-2003 [file 2041-1480-1-S1-S1-S1.doc]

Recommender web service documentation

This section describes how to use the Recommender service. An *alpha* version of the Recommender service is deployed as a REpresentational State Transfer (REST) web service and return results in several formats as text or XML. URLs given in this section are subject to change as the web service is currently being deployed in production environment.

### REST Application Programming Interface (API)

In order to use the Recommender web service REST API a user needs to implement a client application that will send an HTTP POST request to the Recommender base service URL:

http://ncbolabs-dev2.stanford.edu:8080/OBS_v1/recommender1.1/

A simple HTML client to test the Recommender is available here:

<http://obs.bioontology.org/recommender/Recommender1.1.html>

The following parameters are available for the API:

- method – Identifies the method used for recommendation. Each method will request the NCBO Annotator with specific parameters in order to achieve the Recommender heuristics. 4 methods are available:
  - 1 – CR with longestOnly=false
  - 2 – CR+M with longestOnly=true
  - 3 – CR with longestOnly=true
  - 4 – CR+M with longestOnly=true
- output – Identifies the output ranking value considered for ranking ontologies in the results. 4 outputs values are available:
  - score – The sum of the scores of all the annotations generated with concepts from a particular ontology.
  - nb-annotating-concepts – The number of unique concepts annotating the input text for a particular ontology.
  - normalized-score – The ratio between the ontology score and the ontology size.
  - overlap – The ratio between the number of annotating concepts and the ontology size.
- repository – The set of ontologies to consider for recommendation:
  - ncbo – Only the NCBO BioPortal ontologies.
  - umls – Only the UMLS Metathesaurus ontologies.
  - all – Both NCBO & UMLS ontologies.
- text – The text data used for recommendation.
- format – The output format of the results. Ontologies are sorted according to the output parameter. 3 formats are available:
  - asText – Returns the results in text document.
  - asTextSimple – Returns the results in a simplified text document without the output ranking values.
  - asXML – Returns the result in a XML document. Each ontology is represented by a <obs.common.beans.RecommendedOntologyBean> for example:

<obs.common.beans.RecommendedOntologyBean>

<localOntologyID>40644</localOntologyID>

<ontologyName>NCI Thesaurus</ontologyName>

<ontologyVersion>09.07</ontologyVersion>

<virtualOntologyID>1032</virtualOntologyID>

<nbAnnotation>5</nbAnnotation>

<score>42.0</score>

<nbAnnotatingConcept>4</nbAnnotatingConcept>

<normalizedScore>5.656794E-4</normalizedScore>

<overlap>0.0050</overlap>

</obs.common.beans.RecommendedOntologyBean>

Remark: The number of annotations for a particular ontology is also generated however this value cannot be used for ranking.

### User interface

The Recommender is currently being embedded in BioPortal (Figure 1). A simplified user interface allows a user to test some of the Recommender heuristics in a web browser. The two discussed scenarios are available in the user interface (corpus & keyword-based recommendation). An additional parameter allows normalizing the ranking result by ontology size. The methods considered by the user interface are always 1 for corpus-based recommendation and 3 for keyword-based recommendation. The outputs considered are score and normalized-score. Therefore, the user interface addresses the first and third question indentified in Introduction. The user interface allows also selecting the repository to use.

As illustrated in Figure 1, a tag cloud is generated in which the score (or normalized-score) of an ontology is represented by the size of its name in the cloud. When clicking on an ontology name in the cloud, a pop-up window provides the values of each output ranking value for this ontology (Figure 1). A link to the original ontology in BioPortal is also provided when it is appropriate.


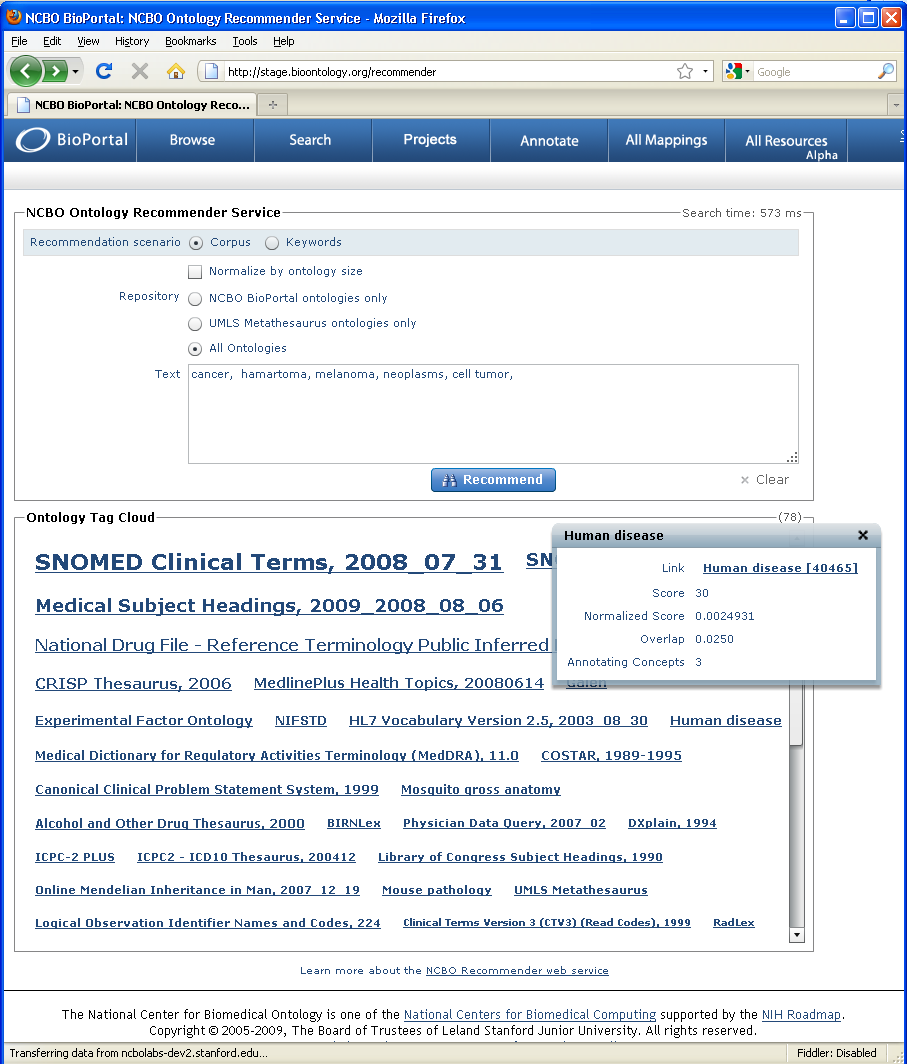


Figure 1 – Recommender web service user interface in BioPortal.
