# Supplementary material for: Building a biomedical ontology recommender web service
Source: J Biomed Semantics. 2010 Jun 22;1(Suppl 1):S1. doi: 10.1186/2041-1480-1-S1-S1 (PMC2903720; doi:10.1186/2041-1480-1-S1-S1)
Supplement: Additional file 2 — Description of data: Contains a detailed comparison of the existing ontology recommendation tools that form the basis of the related work section. File Format: Microsoft Word 97-2003 [file 2041-1480-1-S1-S1-S2.doc]

Detailed comparison of existing recommendation tools

Much work has been done in the semantic web community on evaluating the quality of an ontology for a particular criteria or for a specific application. Existing efforts make a distinction between ontology *recommendation* (also called ontology *selection* or ontology *ranking* in the literature) and ontology *evaluation* approaches. We first explain the distinction, and then present an overview of the recent literature on ontology recommendation. Only a few approaches are applied to the biomedical domain.

## Ontology evaluation vs. Ontology selection

Sabou et al. [Error: Reference source not found] define ontology evaluation and ontology selection as two different things that closely related one another: *Ontology selection is the process that allows identifying one or more ontologies or ontology modules that satisfy certain criteria. The actual process of checking whether an ontology satisfies certain criteria is, an ontology evaluation task*. Indeed, ontology evaluation consists in evaluating an ontology, independently from other ones, following a specific criterion. There are different approaches to evaluate an ontology. Some evaluate the logical correctness of the ontology, some compare metrics, some compare technologies, some compare structure & representation, some evaluate the appropriateness of an ontology for a specific tool or task, some compare ontologies against common benchmarks, some evaluate the appropriateness of an ontology against a corpus of data, some evaluate according to user assessments. The reader might refer to [Error: Reference source not found, Error: Reference source not found, Error: Reference source not found].

Sabou et al. [Error: Reference source not found] illustrate an interesting paradox: few of the existing ontology evaluation techniques are employed by the selection algorithms described after. Most of the approaches for ontology selection are based on some kind of matching (syntactic or semantic) between the query terms (or the processed corpus of data) and the ontology content. Then some measures are applied to the identified concepts and the aggregation of these measures for each concept per ontology usually gives the ranking. The Recommender follows the same general principle. As a consequence, in the following, we only detail ontology evaluation approaches if they have been explicitly used in a tool or method for ontology selection. We review the possible evaluation criteria (actually used) and the properties of the ontology selection approaches that we have identified in the literature.

## Search ontologies approaches

The expression ‘ontology search’ is also sometime used in the literature but is ambiguous as it can have two different meanings: (i) *Search for ontology* which is the process, similar to ontology selection, of finding ontologies corresponding to a given query with or without ranking the results; (ii) *Search in ontology* which is the process of finding concepts or other ontological entity that forms an ontology (e.g., property) across ontologies. Often, the former is based on a method that uses the latter.

Search in ontologies is a feature often present in ontology repository or on the web. BioPortal Search [Error: Reference source not found] and the Ontology Lookup Service (OLS) [Error: Reference source not found] are specifically restricted to biomedical ontologies. They are described in the next section. Swoogle [Error: Reference source not found], Watson, [Error: Reference source not found], OntoSearch [Error: Reference source not found], OntoSearch2 [Error: Reference source not found] and OntoKhoj [Error: Reference source not found] are tools to search in ontologies. Those tools usually crawl the semantic web documents from the web and index them. Then they allow a keyword-based search of their index and they usually return only the ontologies that have the keyword in their class or property names (Watson does actually use also property values). Watson & OntoSearch2 propose a more formal query approach by allowing users to pass SPARQL queries. Swoogle & OntoKhoj use ranking algorithms similar to page rank used by Google to give more importance to ontologies that are highly connected (references to and from). For example, OntoKhoj uses instantiation, sub classes and domain/range links. However, neither OntoKhoj nor Swoogle do use mappings between ontologies which in the biomedical domain are the most frequent way of connecting ontologies (note also that neither BioPortal Search nor OLS use mappings). The Recommender service can use these mappings to discriminate ontologies as previously explained.

Search in ontologies approaches alone are not sufficient for ontology recommendation as they do not provide the final abstraction to say to a user “these are the ontologies you should use” for your data. Those approaches fail addressing the scenarios identified previously (i.e., they cannot deal directly with a large set of keywords or corpus datasets). However, those methods can be used to implement ontology selection workflow as in AKTiveRank [Error: Reference source not found] detailed later.

## Approaches applied to biomedicine

Biomedical ontology repositories like the NCBO BioPortal [Error: Reference source not found] and the EBI Ontology Lookup Service [Error: Reference source not found] usually proposes a search functionality that allows a user to query the repository with keywords. Concept names and synonyms are indexed (e.g., using Lucene) to benefits from the classic search features e.g., exact match or contains. BioPortal proposes also to search in all concepts properties values allowing for example to search with different type of concept identifiers. None of the two mentioned search engines rank their results (as Swoogle and OntoKhoj). Those search in ontologies approaches, even if specialised for biomedical ontologies, failed addressing the need of recommendation previously explained.

In the context of BioPortal, NCBO is experimenting a community-based evaluation approach [Error: Reference source not found]. BioPortal does provide the infrastructure to enable users to give information such as reviews of ontologies for specific projects or notes on ontology concepts or view on ontologies. The underlying questions that the community assessments help to resolve are for examples: Has anyone used this ontology successfully for a task that is similar to mine? Is this ontology currently active (i.e., discussed, used, and maintained)? The Recommender service complements this informal community-based approach with an automatic approach that asserts coverage and connectivity. In addition, we are currently working on implementing different recommendation heuristics that will leverage BioPortal direct and indirect user assessments.

Quite recently, Tan & Mabrix [Error: Reference source not found] describes a list of criteria to consider when selecting an ontology for a biomedical text mining task. The criteria are for example: ontology type (e.g., top-level ontology, domain ontology) supporting technologies (e.g., ontology format, tools available), types of representation accessible in the ontology (e.g., concepts, relations, instances), coverage of the domain, and evaluation in a real system or against a benchmark (e.g., BioNLP, BioCreative). The high level criteria elicited by Tan & Mabrix are indeed useful, and one should say that users have to be aware of those criteria when looking for an ontology for their needs. However, the proposed framework does not provide an automated way to abstract all these criteria and recommend an appropriate ontology. A user has to manually or semi-manually evaluate all of them to decide which ontology to (re)use.

Also in the biomedical domain, Maiga [Error: Reference source not found] proposes a tool for biomedical selection. The semi-automatic tool allows a user to specify task requirements as a query that will be matched against a set of summarized ontologies. Then measures (as described in two sections) based on the coverage between query and ontology content as well as based on the ratio and density of relations in the ontologies are used to rank ontologies. Maiga’s method is therefore more specific than the Recommender but unfortunately only a couple of biomedical ontologies are used where as the Recommender proposes a generic and automatic method over 220 biomedical ontologies.

Alani et al. [Error: Reference source not found] address the scenario where a user wants to search for ontologies relevant for a particular domain (e.g., anatomy). Simple keyword search approaches often do not work in that scenario because of the lack of metadata about ontologies e.g., the term ‘anatomy’ often does not exists in important anatomy ontologies e.g., Foundational Model of Anatomy. Therefore, they propose a corpus-based approach, experimented in the biomedical domain: when looking for ontologies on a particular topic they retrieve, from the web (e.g., Google or Wikipedia), a collection of terms that represent the given domain (e.g., body, brain, skin, etc. for anatomy). The terms are then used to expand the user query and search in the existing biomedical ontologies (for label and synonyms). Their results show an improvement in retrieval results by 113%, compared to the classic search in ontologies approaches that search only for the user query terms (cf. previous section). However, the method does rank the returned ontologies. Alani et al. Address a specific scenario that is not addressed by the Recommender. We do not expand the user query (keyword or corpus) and assume that the given keywords or corpus data are relevant enough. The rest of the Recommender method is similar to Alani et al. method i.e., lookup for label and synonyms but the Recommender does rank the results and allow using mappings in the process. Other corpus or data-based approaches are described hereafter ([Error: Reference source not found, Error: Reference source not found, Error: Reference source not found]) however, Alani et al. and the Recommender distinguish as they are fully automatic (including the corpus selection for Alani et al.).

## Data driven approaches

Data driven approaches are interesting as they allow considering the first scenario identified in introduction: which ontology ‘fit’ the best for this data? Brewster et al. [Error: Reference source not found] was the first data driven ontology evaluation approach which consisted in evaluating the appropriateness of an ontology against a corpus. The method consists in (i) identify the keywords & terms of the corpus; (ii) expand the terms using a knowledge base such as WordNet; (iii) map back the indentified terms to the ontology concepts.

OntoSelect [Error: Reference source not found] is also a corpus-based ontology selection approach and their method is similar to [Error: Reference source not found]. OntoSelect is an ontology library that helps selecting an ontology for annotation where a given web page is considered as the corpus. OntoSelect’s ontology selection method also relies on how well an ontology is connected to other ontologies (using importation mechanism) as well as on structure measures (as described in next section). In [Error: Reference source not found], the authors proposed a data driven selection approach based on description logic. They use reasoning on ontology module to go beyond simple syntactic approach to achieve topic coverage.

There are two main risks in those approaches: the recommendation is dependent on both the identification of keywords that can miss some important terms and the expansion (in the case of [Error: Reference source not found] and [Error: Reference source not found]) of those keywords than can introduce noise. The Recommender avoids those two drawbacks and gives more importance to concept recognition step (using preferred name and synonyms) assuming the input data is representative enough. The scoring algorithm of the Recommender takes into consideration term frequency as suggested by these methods (in the keyword extraction step).

## Measures based and other miscellaneous approaches

AKTiveRank [Error: Reference source not found] was the first tool for ontology recommendation that ranks candidate ontologies following several measures based on the ontology structure. The task of selecting candidate ontologies is delegated to an external ontology search engine. AKTiveRank takes keywords as input, and queries Swoogle for the given keywords in order to extract candidate ontologies. Then AKTiveRank applies measures based on the coverage (i.e., finding concepts matching with class and property names) and on the structure of the ontologies to rank them. AKTiveRank is not restricted to any particular domain but, as many other tool mentioned, only deals with ontologies compliant with semantic web standards (Ontology Web Language (OWL) and Resource Description Framework (RDF)). This is a major issue in the biomedical domain where many ontologies are in the OBO or RRF format. The Open Biomedical Ontology format is the format used by the OBO Foundry initiative [Error: Reference source not found]. The Rich Release Format (RRF) is the format of ontologies embedded in the UMLS Metathesaurus [Error: Reference source not found]. Moreover, not being restricted to the biomedical domain prevents AKTiveRank to use abstractions such as the ‘synonym’ property provided by BioPortal or UMLS which significantly enhance the matching step.

OntoQA [Error: Reference source not found] proposes another set of measures to evaluate the quality of an ontology. Whereas AKTiveRank measures are at the ‘concept level’ (i.e., measures are applied on (or between) concepts after the first step matching, OntoQA measures are at the ‘ontology level’ and are independent of any previously identified concepts. For instance, OntoQA looks at the diversity of relations in an ontology schema or the average number of attributes per concepts or the distribution of concepts across the hierarchy. OntoQA originality is that it proposes measures to evaluate the quality of the knowledge based associated with an ontology schema (i.e., instances).

CombiSQORE [Error: Reference source not found] proposes a method similar to [Error: Reference source not found]. Assuming a ‘semantic query’ (decomposed in sub queries) as input, the tool expands the query with WordNet and then uses the result of ontology search engines i.e., Swoogle and Watson to retrieve ontologies. Then measures are applied to rank ontologies based on the similarity between the sub-queries and the ontologies. An originality of CombiSQORE measures that is similar to the Recommender is the use of the ontology size to compare ontologies together.

Hong et al. [Error: Reference source not found] propose a non automatic interactive approach for selecting an ontology that fits the best a formalized domain. This method is similar to the ontology evaluation approach based on gold standard.

WebCORE [Error: Reference source not found] (similarly to [Error: Reference source not found]) introduces another criterion which is the collaborative assessments provided by users in an ontology library platform. They work aims to enhance ontology selection by combining automatic evaluation technique with explicit users’ opinions and experiences. Their method consist in a first step in taking a set of initial terms and expanding it through WordNet and then use classic ontology coverage metric to elicit a list of candidate ontology. In a second step, this list is ranked taking into consideration previous feedback and evaluation of the users. The authors are mainly interested in the use case of selecting (non automatically) an ontology for re-use. They do not pay attention to the scenario where automatic quick recommendation is needed.

Sabou et al. [Error: Reference source not found] present a method for ontology selection that has two interesting originalities: (i) a semantic match that goes beyond the simple syntactic match when doing the matching between keywords and concepts; (ii) the identification of the appropriate ontology combination that will address a complete coverage of the query terms. The method also uses WordNet for query expansion if necessary. However, the method is not fully implemented or available as a service. We note that the Recommender service, in the keyword scenario for which this is relevant, does not provide the smallest set of ontologies that offers 100% coverage of the given keywords. This is another abstraction on the Annotator results that could be interesting to look at.
